# 5G-enabled contactless multi-user presence and activity detection for independent assisted living

**DOI:** 10.1038/s41598-021-96689-7

**Published:** 2021-09-02

**Authors:** Aboajeila Milad Ashleibta, Ahmad Taha, Muhammad Aurangzeb Khan, William Taylor, Ahsen Tahir, Ahmed Zoha, Qammer H. Abbasi, Muhammad Ali Imran

**Affiliations:** James Watt School of Engineering, University of Glasgow, Glasgow, G12 8QQ UK

**Keywords:** Electrical and electronic engineering, Biomedical engineering

## Abstract

Wireless sensing is the state-of-the-art technique for next generation health activity monitoring. Smart homes and healthcare centres have a demand for multi-subject health activity monitoring to cater for future requirements. 5*G*-sensing coupled with deep learning models has enabled smart health monitoring systems, which have the potential to classify multiple activities based on variations in channel state information (CSI) of wireless signals. Proposed is the first 5G-enabled system operating at 3.75 GHz for multi-subject, in-home health activity monitoring, to the best of the authors’ knowledge. Classified are activities of daily life performed by up to 4 subjects, in 16 categories. The proposed system combines subject count and activities performed in different classes together, resulting in simultaneous identification of occupancy count and activities performed. The CSI amplitudes obtained from 51 subcarriers of the wireless signal are processed and combined to capture variations due to simultaneous multi-subject movements. A deep learning convolutional neural network is engineered and trained on the CSI data to differentiate multi-subject activities. The proposed system provides a high average accuracy of 91.25% for single subject movements and an overall high multi-class accuracy of 83% for 4 subjects and 16 classification categories. The proposed system can potentially fulfill the needs of future in-home health activity monitoring and is a viable alternative for monitoring public health and well being.

## Introduction

In recent years 5G-based technologies, pointing at seamless integration of the world into a single system, have been making significant innovations in numerous real-world implementation and thus introducing significant commercial possibilities for several fields such as healthcare and energy conservation. In healthcare, 5G is already creating new opportunities for areas such as medical imaging, data analysis, elderly care, diagnostics, and prognostication due to its ultra low latency, Massive-Machine Type Communication (mMTC), intelligent management and handling of Big Data^1^. However, Human Activity Recognition (HAR) has received increasing attention due to numerous applications in fields such as security, health care and smart utilisation of resources, but has not been introduced with 5G-based technologies. HAR and classification not only brings numerous benefits to elderly care^2,3^ but also is a basic key in providing higher-level appropriate information such as positions, activities, occupancy, and identities in an indoor environment that can be useful for health care, source utilisation, security, and in energy saving.

The risks of high carbon emissions on the environment have increased the need for solutions, across all societal domains, to achieve carbon neutrality. One of the key points to be considered is reducing energy waste, that is, consuming energy only when and if needed^4^. Presence and activity detection systems can play a vital role in this and can complement Internet of Things (IoT) use cases for smart cities, such as the wireless electricity data logger introduced in^5^, to control in-home energy consuming systems such as lights, appliances, and so on.

Several studies in the literature have discussed and evidenced the relationship between building occupancy and energy consumption. For instance, a study was reported in^6^ showing that forecasted energy consumption can be skewed by up to 300% if occupancy was not accounted for. Another study^7^ confirmed this by showing that actual consumption could be up to three times greater than the simulation and one of the factors contributing to this big gap is occupants’ behaviour and their numbers^8,9^. Thereby, it is fair to say there is a strong correlation between occupancy and levels of energy consumption^10^, which makes a compelling case for occupancy monitoring systems and their positive impact on the environment.

In recent years, the attention towards contactless monitoring/detection of human activity has greatly increased and a lot of promising research work has been reported in this area. Contactless monitoring/detection of human activity employs techniques such as ultrasonic^11^, vibration^12–14^, smartphone^15^, radar^16^ and Radio Frequency (RF) sensing. The primary disadvantages of using ultra sound, vibration and radar sensing systems are the cost and set-up of additional equipment. The use of smartphones can be beneficial due to higher powerered mobile devices becoming more available however they cannot be considered fully contactless as users must carry their device all the time. The use of RF-sensing addresses these disadvantages as RF signals are already present within the home with the use of Wi-Fi and is completely contactless. The methods based on RF-sensing^17,18^ are gaining considerable popularity due to their privacy-friendly feature and for being contactless in comparison with conventional systems either based on cameras or wearable sensors^19–22^ and thus an introduction of 5G-based HAR and classification will certainly revolutionise this area of research.

Generally, the existing HAR systems based on RF-sensing differ from each other on basis of various factors such as hardware specifications, operating radio frequency, classification techniques, number of activities to monitor and number of subjects performing those activities. Available HAR systems either make use of Received Signal Strength Indicator (RSSI)^23–25^ or Channel State Information (CSI)^26–28^, for the purpose of activity recognition. However, studies, such as^29^, suggest that CSI is more robust to the complex environments as CSI is fine-grained and measured per Orthogonal Frequency-Division Multiplexing (OFDM) from each packet while RSSI contains the coarse information that is a single value per packet. This makes CSI more stable to be used for localisation and activity recognition. Furthermore, due to recent advances CSI based systems do not require the user to carry wearable devices and do not depend on lighting. Also, it can detect human behaviour through the wall and complex situations. Making them a good alternative for RSSI based recognition systems.

Recently, many CSI and machine learning-based approaches have been proposed to accurately detect and count people in a given coverage area of a Wi-Fi network. For example, in^26^ the authors proposed a Wi-Fi-based multi-user gesture recognition method to recognise 6 different gestures performed by 10 volunteers. A similar approach based on CSI has been proposed in^30^ for crowd counting. Both of these studies make use of 1 transmitter (Tx) and 3 receiver antennas (Rx) and use the intel wireless network card. However, Network Interface Card (NIC) usually presents numerous limitations. For instance, the transmitter used in those system sends a group of 56 subcarriers, however, the NIC only reports 30 subcarriers, nearly 46% of the information lost in this case. The authors in^31^ exploit the CSI of Wi-Fi to detect multiple subjects’ activities. This system applied a three-phase system Wi-multi to recognise three different activities performed in parallel, including in a noisy environment. They achieved an accuracy of around 96% using 2 Tx and 3 Rx antennas. In another study^32^, the authors presented a method of crowd counting based on CSI extracted from a Wi-Fi device with 2 Tx and 3 Rx antennas. The proposed method exploits both amplitude and phase information of CSI signal to perform crowd counting only and does not deal with activity recognition. The work presented in^33^ used CSI data of Wi-Fi device with 2 Tx and 3 Rx antennas to detect sixteen different exercises performed by ten different subjects. However, the authors did not deal with the activities performed in parallel. 5G has been used in^34,35^ to detect breathing rates of people suffering from diabetic keto acidosis (DKA). The proposed system makes use of C-band sensing operating at 4.8 GHz. C-band communication is an important component of 5G communication. 4.8 GHz was also used in the work of^36^ to detect the Freezing of Gait (FOG) which is an abnormal gait pattern that accompanies Parkinson’s disease. This work used the 5G spectrum to detect and classify FOG by sensing the human movements. The work of^37^ made use of the 5G frequency of 3.45 GHz to detect human presence and walking speed using RF-sensing.

The brief overview of the literature, presented above, suggests some important insights. For example (i) most of the studies involve CSI data extracted from commercial Wi-Fi devices such as NICs that report a limited number of data subcarriers and causes a loss of information and thus adversely affect the classification performance. Moreover, NICs are primarily used for networking functions, hence it’s reliability can be significantly impacted if it was simultaneously utilised for sensing. Hence, to ensure high accuracy and reliability, this paper presents a system that makes use of Universal Software Radio Peripheral (USRP) devices, where the hardware can be controlled over the software, and parameters such as the number of subcarriers can be controlled and fully utilised. The USRP platform also allows to modify the transmitted and received power and the operating frequency swing, through which the system could be easily configured to operate in the 5G-band (< 6 GHz). Furthermore, the ease in implementation of signal processing algorithms and the ability to reuse hardware encourages researchers to choose USRP devices for their applications. (ii) All of the above-mentioned studies use more than one Tx and Rx antennas to capture the maximum information, whereas in this paper experimental results show that the proposed method achieved even better performance in recognising multiple activities performed in parallel with one Tx and Rx antenna.

The remainder of this paper is structured as follows: In “Methodology and framework” the details of the proposed methodology, tools, and experimental design aspects are described in details; “Results and discussion” goes on to detail the conducted experiments, the obtained results, and their discussion; and finally, in “Conclusions and future work” the paper is concluded.

**Figure 1:**
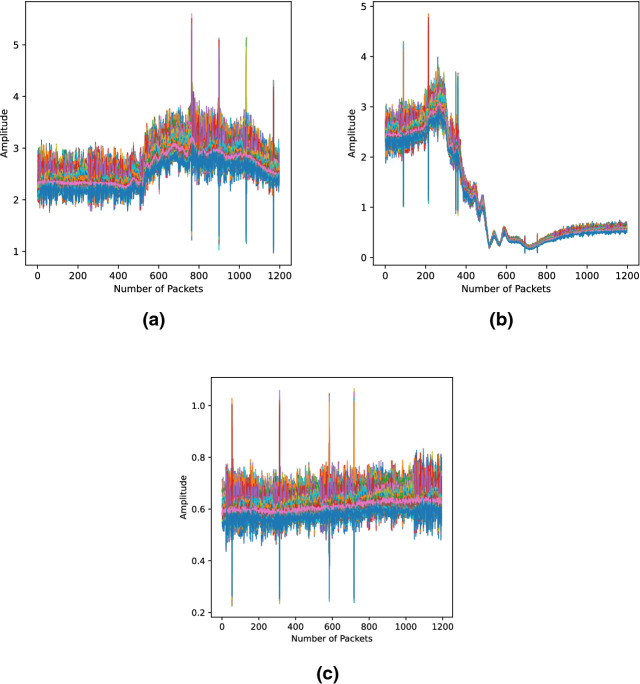
Channel state information patterns of activities performed at 5 GHz (Wi-Fi frequency) and 3.75 GHz (5*G* Frequency); (**a**) Standing Activity at 5 GHz, (**b**) Standing Activity at 3.75 GHz, (**c**) Empty Room at 5 GHz.

*Contributions and study motivation* In this paper a novel 5G-enabled presence and activity detection system, of multiple subjects, is presented. The proposed RF-sensing system was designed to operate in the 5G frequency band, particularly at 3.75 GHz. The ultimate goal is to enable the incorporation of 5G-based non-invasive in-home activity monitoring systems in our community to maximise the utilisation of the opportunities offered by 5G and its enabling technologies. The contributions in this paper can be summarised to the following with the motivation behind each described after every point:

1. Presence and activity detection of multiple subjects performing different activities in parallel. Activity recognition for single subjects has been greatly explored by several studies in the literature^38–42^. The research team presenting this paper, have led several studies on activity recognition using software defined radios^43–45^, which motivated them to take it to the next level and conduct one to explore the capability of the technology to do so for multiple subjects. By utilising RF-sensing technology and Artificial Intelligence (AI), a single system is presented that can simultaneously monitor occupancy, that is count the number of people in a room, and detect the parallel activities of all subjects. The contribution here has two folds, the first is accounting for a combination of three different activities occurring in parallel, amongst four different subjects. Secondly, the variation introduced in the training data by introducing intra-class variation, that is, training the system to classify the same activity or combination of activities performed in different positions within the room and by different test subjects, as the same class. This was performed to strengthen the machine learning model and improve its detection accuracy.
2. 5G-enabled sensing, that is, the proposed RF-sensing system was designed to operate in the 5*G*-band, particularly at 3.75 GHz. To the best of the authors’ knowledge, the use of 5*G* for multi-user sensing applications has not been considered elsewhere in the literature. The motive and ultimate goal for the use of this frequency is to utilise 5G technology with its high data rates and ultra-low latency capabilities in developing real-time non-invasive activity and presence detection systems for assisted living. Moreover, primary findings from the experiments conducted in this paper have shown that the CSI, reflecting activities performed by a test subject, captured using the 5G frequency 3.75 GHz are much more evident in pattern compared to those captured at the Wi-Fi frequency. To confirm this, the experimental setup, presented later on in “Methodology and framework”, was used to collected CSI samples at both frequencies for the “Standing Activity” and for “Empty Room” at the Wi-Fi frequency 5.00*GHz*. All three captures can be found in Fig. 1, where Fig. 1a and 1b, are of a “Standing Activity” captured at 5 GHz and 3.75 GHz, respectively. Whilst Fig. 1c, represents a capture of an “Empty Room”, at 5.00*GHz*. The evident pattern in the captured CSI, such as that in Fig. 1b is more likely to increase the accuracy of classification, compared to that in Fig. 1a, especially in a real-time system when massive amounts of data are captured and processed.
3. The data sets collected during the course of the experiments, presented in this paper, are made publicly available through this link^46^. The lack of a comprehensive data set for this type of activities has motivated the research team to make the data available and benefit the wider community with this rich data set that covers a wide range of activities. Researchers can use the data set to apply different processing techniques, replicate the experiment and collect more data for bench marking. The online data set contains a total of 1777 samples divided amongst 16 classes, as detailed later on in “Experimental design”.

## Methodology and framework

**Figure 2:**
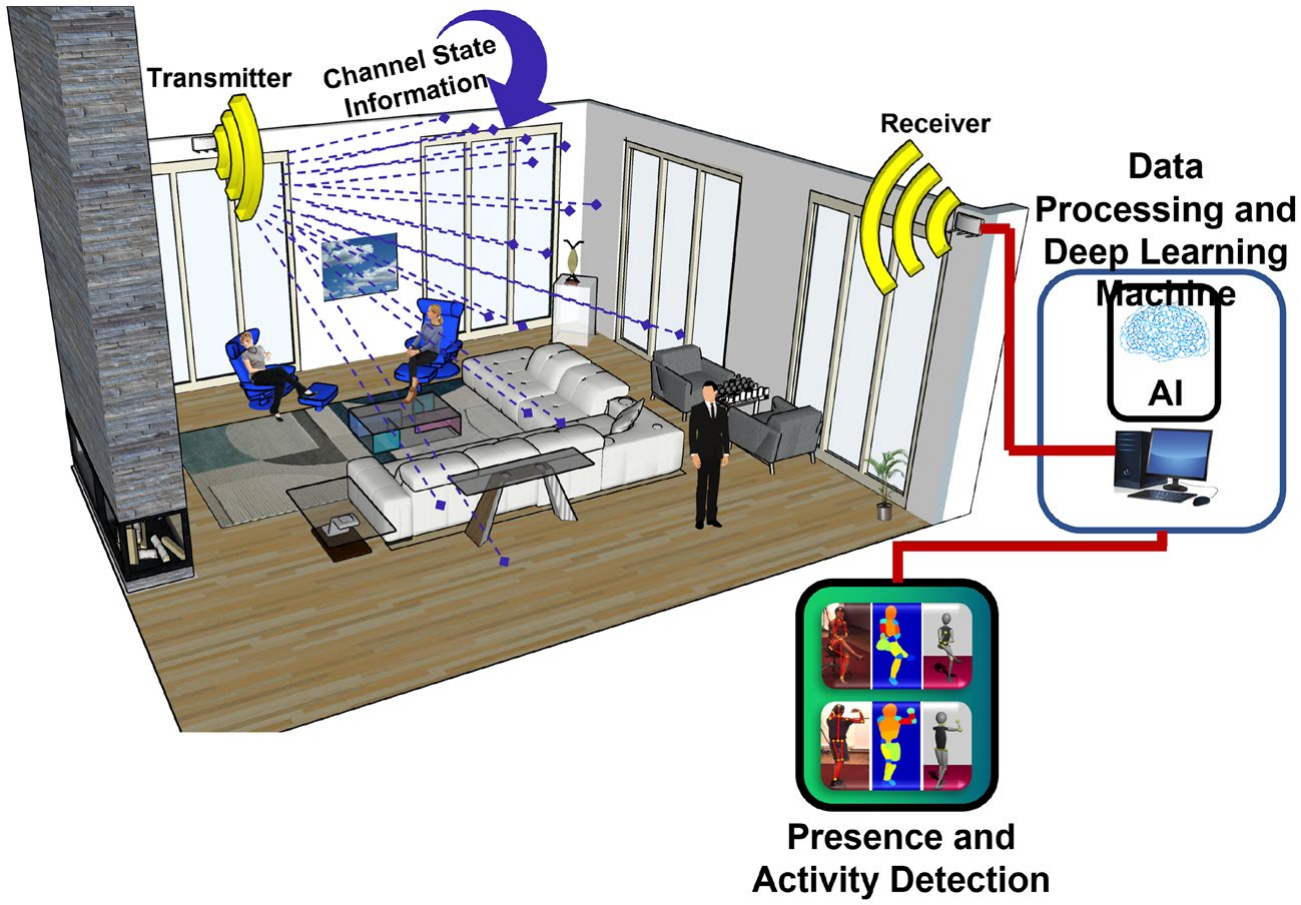
System concept diagram showing the contactless sensing system for multi-users using wireless signals.

This section details the methodology and framework adopted to conduct the experiments and achieve the reported results. It starts by presenting the specifications of the hardware design stage, followed by a detailed outline of the experimental design stage including experimental variables, data collection, data processing, system training, and testing. The system conceptualisation and main building blocks are presented in Fig. 2.

### Hardware design specifications

The experiments conducted and reported in this paper utilised two USRPs devices each equipped with the VERT2450 omnidirectional antenna. One USRP is used as the transmitter and the second USRP is used for the receiver. Each USRP is connected to a separate PC that uses the Intel(R) Core (TM) i7-7700 3.60 GHz processors and has a 16 GB RAM. The system makes use of virtual machines to provide the Ubuntu 16.04 operating system. On the Ubuntu virtual machines, Gnu Radio is used to communicate with the USRPs. Gnu Radio allows for the creation of flow diagrams for the USRP function to be carried out. These flow diagrams can then be converted to python scripts. One python script is created to transmit data and another script is configured to receive the data from the transmitter. The transmitter transmits random numbers between 0 and 255 using OFDM. The receiver side is configured to receive the transmitting signal from the transmitter USRP. The script ran on the receiver side then outputs the CSI complex number to the terminal. This output is then processed to extract the amplitude values from the CSI complex numbers. The system’s main configuration parameters are shown in Table 1.

**Table 1:** Software configuration and parameters’ selection.

Parameter	Value
Platform	USRP X300/310
OFDM subcarriers	51
Operating frequency	3.75 GHz
Transmitter GAin (dB)	70
Receiver gain (dB)	50

### Experimental design

**Figure 3:**
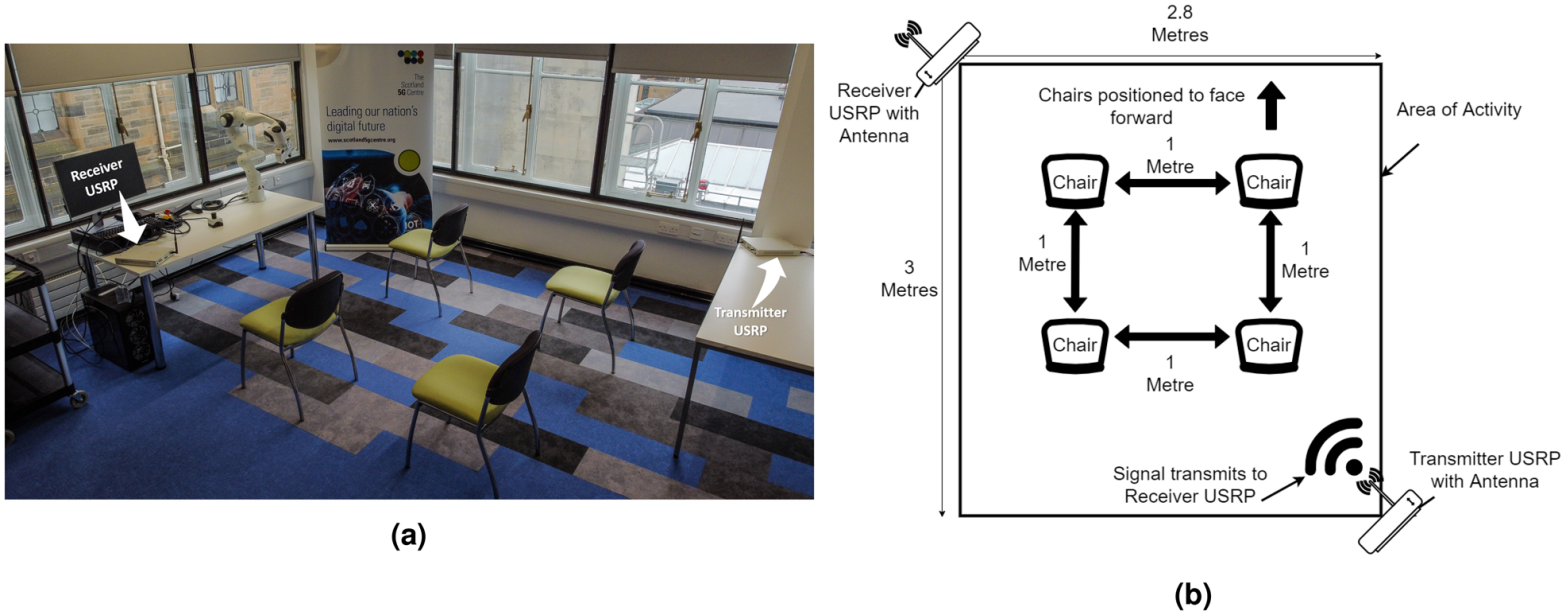
Experimental setup for capturing activities using 5G frequency.

To validate the effectiveness of the proposed framework, various experiments have been performed in a rectangular $2.8\times 3 m^2$ activity area as shown in Fig. 3. The two X Series USRP devices for the transmission and the reception of CSI signals have been installed in the two corners facing each other. To capture maximum intra-class variation for all the activities that include sitting, standing and walking, the subjects have kept changing their positions randomly in the prescribed activity area while keeping a 1*m* distance among themselves during the course of multiple experiments. This is to simulate a small setting such as a care home with a limited number of people. Currently the focus of the experiment is to accommodate 4 people. Part of the future work will seek to increase this number.

**Figure 4:**
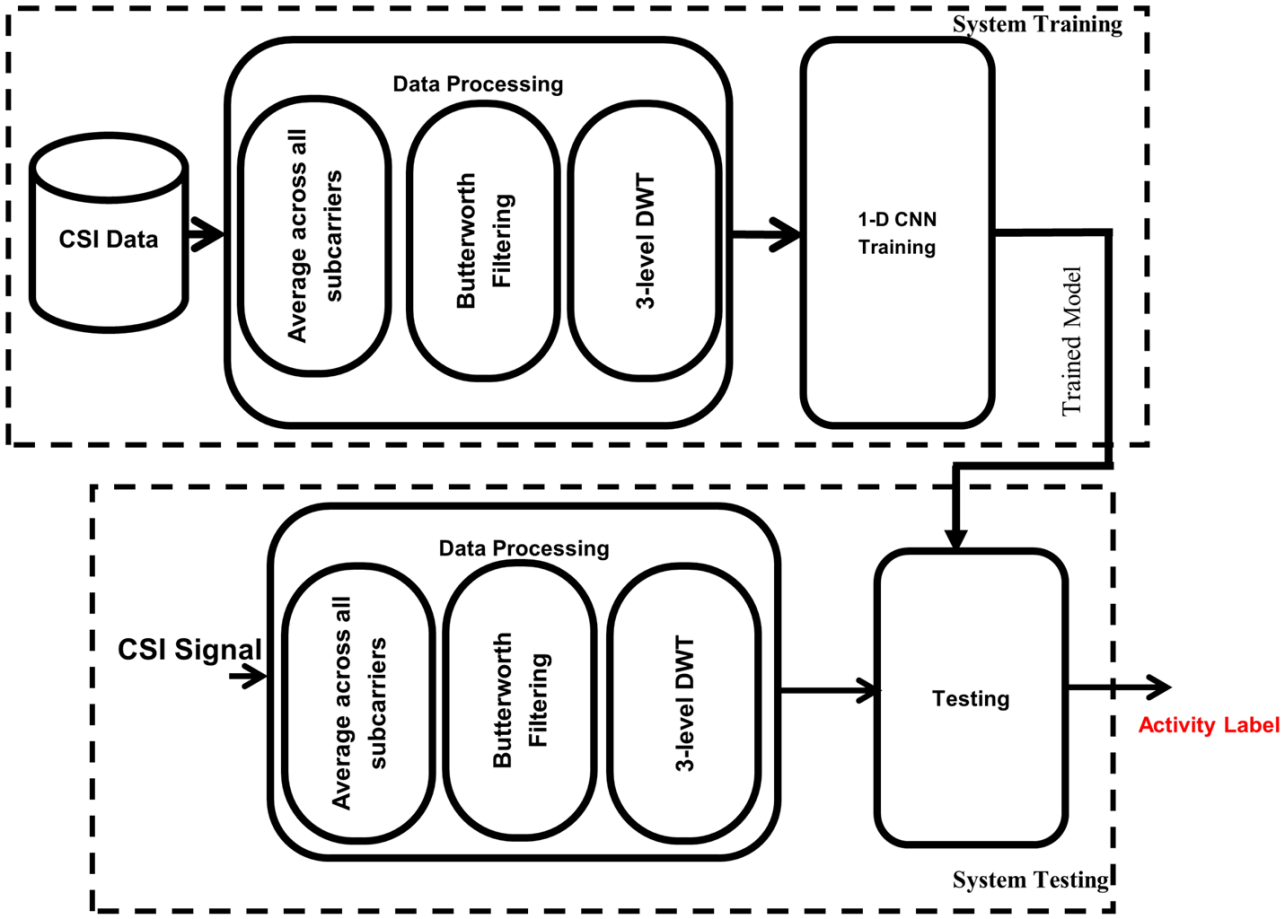
Signal flow diagram for multi-user activity classification.

Furthermore, the proposed deep learning-based classification methodology to recognise multi-user activities is comprised of two major modules: (i) System Training and (ii) System Testing, as depicted in a high-level signal flow diagram shown in Fig. 4. The system training module is based on an offline process that involves already acquired and preprocessed CSI samples data set to train the 1-D Convolutional Neural Network (CNN). The system testing is performed in an online setting in which an input CSI data sample, after all necessary preprocessing is performed, is classified as one of the human activities. A detailed description of the two modules is provided in subsequent sub-sections.

#### System training

The system training module of the proposed classification architecture is mainly based on the three major components (see Fig. 4): (i) Data collection, (ii) Data processing and (iii) 1-D CNN. The CSI data acquisition was performed using the setup shown in Fig. 3. To prepare the captured CSI data for subsequent classification, tasks are done in the data collection and data processing modules, respectively. Whereas the third component deals with the training and learning of the 1-D CNN model. A detailed discussion on each of these components is presented in the following sections.

*Data collection* The data collection for the proposed system training involved five steps with four volunteers each performing three different activities, “Sitting”, “Standing”, and “Walking” in a lab environment, with the setup shown in Fig. 3b. The setup was replicated in two different lab environments as means of introducing different clutter levels which increases the variability of the data and further strengthens the model. Nevertheless, the data for a particular class collected in both environments were treated as the same data set, that is, clutter level due to environment was not a measured variable in the conducted experiments.

As with any experiment, there were some fixed attributes as well as variable ones. For the experiments presented in this paper, the fixed attributes included (1) The hardware and its configuration (2) The data processing and deep learning techniques and (3) The experimental setup, shown in Fig. 3. The experimental variables included (1) the number of subjects (2) The subject identity, in the data collection for one and two subjects and (3) The location of the performed activity, that is, for example, for one subject performing “Sitting Activity” it was performed in different chair locations, as per Fig. 3. The first variable was measured as will be highlighted in the remainder of this section and in the results, whilst the second and third variables were only utilised to introduce maximum intra-class variation in the collected data. All the data was collected over a calendar week, with a random number of samples collected for all 16 classes in every day of the week to ensure the repeatability of the data over different days.

The “Sitting” and the “Standing” activities are representations of the action of performing these activities and not the posture or the position of the person in the sitting/standing state. Moreover, while capturing the activity data the volunteers were not stressed or forced to keep the upper body still and static so both “Sitting” and “Standing” activity data included the small variations of upper body.

In the first data collection step, the CSI data for a single subject were collected separately for all three activities, that is “Sitting”, “Standing”, and “Walking”, where a total of 420 samples were collected. To introduce maximum variation into the data set, three different subjects participated in this data collection phase. Each subject contributed equally to the data collection, that is, each participant was involved in the collection of 140 samples, divided among the three activity classes. For each CSI data sample, 1200 packets in three seconds are transmitted.

The second step involved data collection for two subjects performing the above mentioned three activities, where a total of 400 samples were collected, as highlighted in Table 2. The same three participants were involved in this data collection stage, with equal contributions from each, that is, each participant was involved in the collection of at least 33 samples for each of the four classes identified for this stage of data collection.

**Figure 5:**
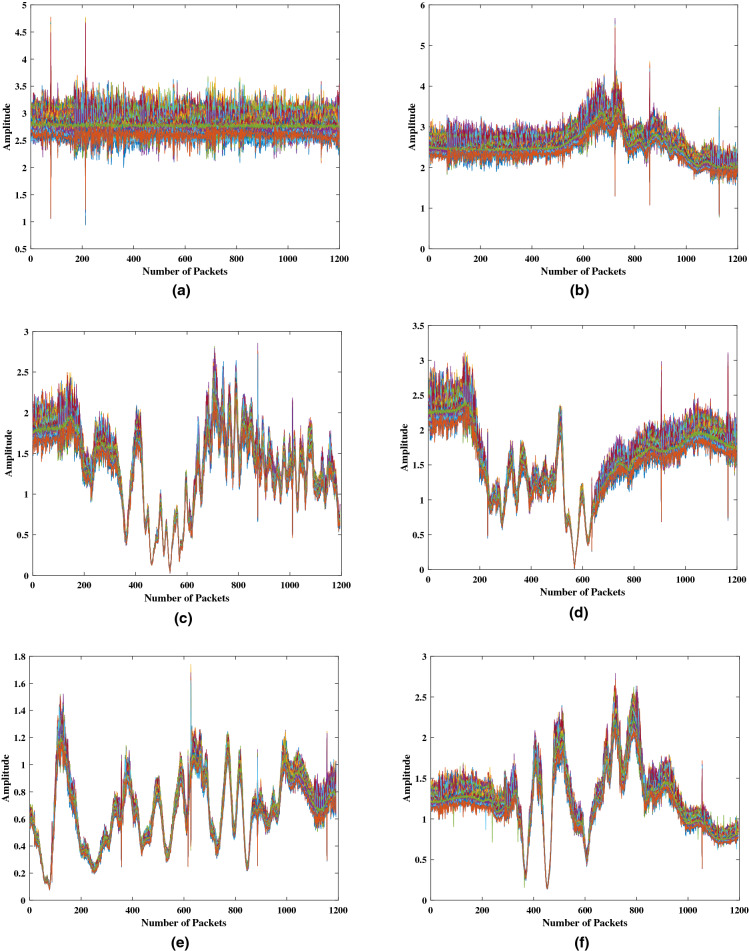
CSI data samples representing various activity classes: (**a**) Empty, (**b**) 1-Subject Sitting, (**c**) 2-Subjects (1 Walking and 1 Sitting), (**d**) 3-Subjects (1 Sitting and 2 Standing), (**e**) 3-Subjects Standing and (**f**) 4-Subjects Standing.

In the third and fourth data collection steps, three and four participants were recruited to participate in collecting the data for three and four subjects performing activities, as outlined in Table 2. The participants recruited for these data collection stages were fixed throughout. In these two steps, 540 and 300 data samples were collected, respectively. In addition to the activity data, 117 data samples were collected for the class “Empty” which represents the status of the room when the subjects are absent from it. All the 16 classes are shown in Table 2. Some of the data samples representing different activities are shown in Fig. 5. The inter-class variation in the data samples of different activity classes is obvious and can be exploited in subsequent classification process to get better results.

**Table 2:** Number of subjects and activities performed.

No. of subjects	Class number	Class/activity	Number of data samples
0	1	Empty	117
1	2	1 sitting	140
	3	1 standing	140
	4	1 walking	140
2	5	1 sit + 1 stand	100
	6	1 walk + 1 sit	100
	7	2 sitting	100
	8	2 standing	100
3	9	1 sit + 2 stand	120
	10	1 walk + 2 sit	120
	11	2 sit + 1 stand	100
	12	3 sitting	100
	13	3 standing	100
4	14	4 sitting	100
	15	4 standing	100
	16	2 sit + 2 stand	100

**Figure 6:**
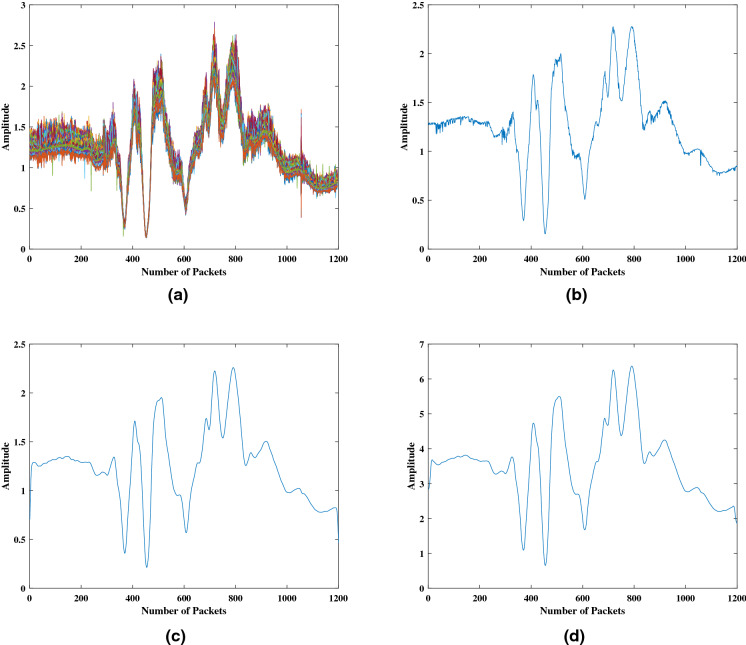
Data processing: (**a**) Raw data sample, (**b**) Averaged across all 51 sub-carriers, (**c**) Butterworth low pass filtering and (**d**) Approximation coefficients of a 3 level Discrete Wavelet Transform.

*Data processing* CSI for one transmitter and one receiver antenna forms a matrix that contains frequency responses of all $N=51$ subcarriers as shown in Eq. (1),

1 $$\begin{aligned} H = [H_1(f),H_2(f), \ldots , H_N(f)]^T, \end{aligned}$$

here frequency of each subcarrier $H_i$ can be represented as

2 $$\begin{aligned} H_i\left( f\right) = |H_i\left( f\right) | e^{j \angle H_i(f)}, \end{aligned}$$

where $|H_i(f)|$ and $\angle H_i(f)$ are the amplitude and phase responses of the *i*th subcarrier. Each of these subcarrier response is related to system input and output as given in Eq. (3),

3 $$\begin{aligned} H_i(f)=\frac{X_i(f)}{Y_i(f)}, \end{aligned}$$

where $X_i(f)$ and $Y_i(f)$ are the Fourier transforms of input and the output of the system.

In general, the acquired CSI data is masked due to the high-frequency environmental noise and multipath propagation of CSI signal. Therefore, to denoise the data and to prepare it for the subsequent training of the 1-D CNN the data is passed through the following data processing steps:

- In the first step, each CSI data sample is averaged across all 51 subcarriers, see Eq. (4), to get one averaged data sample to be used in subsequent processing,
  4 $$\begin{aligned} x_{i}=\frac{1}{J} \sum _{j=1}^{J} y_{i j}, \end{aligned}$$
  where $x_{i}$ is the *i*th data sample that represents the average across corresponding subcarriers $y_{i j}$ for $(j=1,2, \ldots 51)$.
- Afterwards, a Butterworth lowpass filter of order $n=4$ is used to smooth out and to remove small variations from each averaged data sample $x_{i}$.
- Lastly, a discrete wavelet transform (dwt) at level 3 with a Haar basis function is applied to get the approximation coefficients $A_i$ for each of the smooth data samples $s_{i}$. The approximation coefficients represent the output of the lowpass filter in dwt, therefore it further helps in reducing the noise. Mathematically, the convolution and downsampling process involved in the wavelet decomposition for all three levels is represented as follow:
  5 $$\begin{aligned} A_i^0[m]= & {} \sum _{k=0}^{M-1}s_{i}[k] \times g[2m-k], \qquad for \qquad m = 1,2,\ldots ,M. \end{aligned}$$
  6 $$\begin{aligned} A_i^1[t]= & {} \sum _{k=0}^{T-1}A_{i}^{0}[k] \times g[2t-k], \qquad for \qquad t = 1,2,\ldots ,T=\frac{M}{2}. \end{aligned}$$
  7 $$\begin{aligned} A_i^2[u]= & {} \sum _{k=0}^{U-1}A_{i}^{1}[k] \times g[2u-k], \qquad for \qquad u = 1,2,\ldots ,U=\frac{M}{4}. \end{aligned}$$

where *g*[*k*] for $k = 1, 2, \ldots ,K$ is the lowpass filter of length *K* for each decomposition level, $s_{i}[m]$ for $m = 1, 2, \ldots , M$ is the smooth signal of length *M* after applying Butterworth lowpass filter, and $A_i^l$ for levels $l = 0, 1, 2$. is representing the approximation coefficient of three levels of dwt.

Figure 6 shows a raw data sample and the results obtained after each of the data processing steps. Once all the samples are processed, data set is ready to train the 1-D CNN.

**Figure 7:**
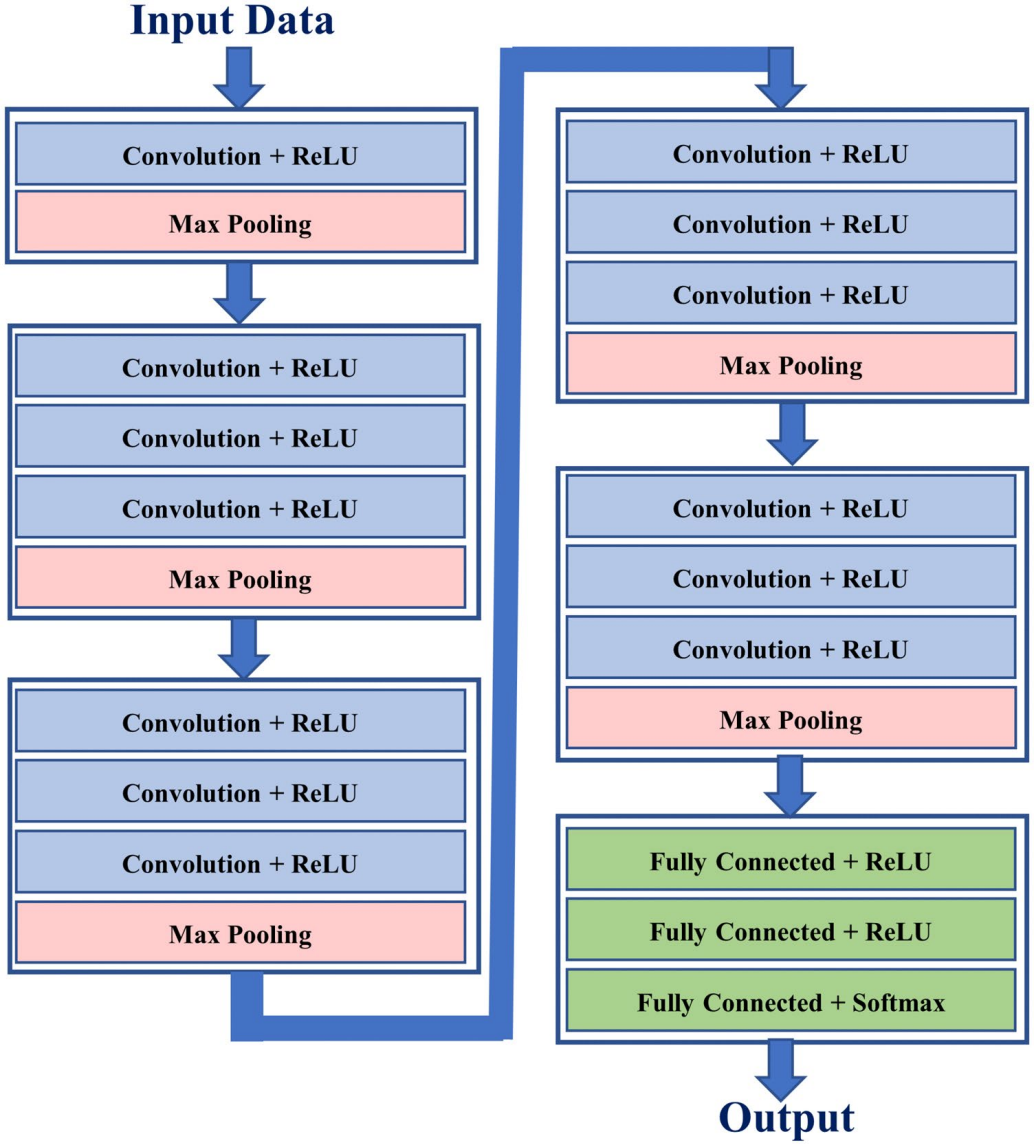
Architectural diagram of the proposed 1-D CNN.

*1-D Convolutional Neural Network* The CNN^47^ is one of the most widely used Deep Neural Network (DNN) for the purpose of pattern classification from both 2-D images and 1-D data signals. 1-D CNNs that have been recently introduced and got a lot of popularity in dealing with the classification problems related to 1-D signals^48^. Motivated by its high accuracy rates in the classification applications^49^, in this paper, we have also adopted a 19 layers 1-D CNN to recognise multiple human activities performed by multiples subjects in parallel. The purposed 1-D CNN structure is comprised of 6 blocks of convolutional layers and one block of fully connected layers. Out of 6 blocks of convolutional layers, the first block contains one convolutional layer and one pooling layer whereas each of the remaining 5 blocks contains 3 convolutional layers and one pooling layer. Finally, the block of fully connected layers contains 3 layers. The complete architecture of the network is shown in Fig. 7 whereas the detail of various parameters used is given in Table 3. Once all the data is preprocessed and the 1-D CNN is trained the trained model is stored for the subsequent testing phase to classify incoming test CSI signal in one of the activity classes.

**Table 3:** Parameter values used in the 1-D CNN.

	Parameter		Value
Architecture	Block-1	1D conv layers	1
		Kernels for each layer	8
		Kernel size	5
	Block-2	1D conv layers	3
		Kernels for each layer	16
		Kernel size	5
	Block-3	1D conv layers	3
		Kernels for each layer	16
		Kernel size	5
	Block-4	1D conv layers	3
		Kernels for each layer	32
		Kernel size	5
	Block-5	1D conv layers	3
		Kernels for each layer	64
		Kernel size	5
	Block-6	1D conv layers	3
		Kernels for each layer	64
		Kernel size	5
	Block-7	1D conv layers	480
		Kernels for each layer	40
		Kernel size	16
Training	Learning Algorithm		Adamax
	Batch size		32
	Epochs		100
	Learning rate		0.001

### System testing

The second phase of the proposed methodology is the system testing that involves the following steps:

- In the first step, the CSI signal obtained from the USRPs is first processed by a Butterworth filter to make it smooth by removing small variations. Then the smooth signal is passed through Wavelet Transform, as described in the previous section, to get approximation coefficients at level three.
- In the second phase, the trained 1-D CNN model is used to classify the processed signal into one of the activity classes.

### Ethics

The current study was approved by the University of Glasgow’s ethics board (application number: (300190109). All experiments were performed in accordance with relevant guidelines and regulations and informed consent was sought from all participants prior to conducting the experiments.

## Results and discussion

The proposed human activity monitoring system was evaluated using two different types of experiments. The first set of experiments focused on determining the accuracy of the system to count the number of people performing particular activities in a room (see Phases 1 and 2 in Table 4). Whilst the second set was conducted to measure the system’s accuracy in identifying different postures/activities of multiple people in the same room. Both types of experiments were performed under a train-and-test split strategy with 80% of the random data was considered as training data while the remaining 20% was taken as the testing data. Furthermore, each experiment was repeated 10 times to get the average accuracy rates for both sets of experiments. The proposed 1-D CNN architecture consists of 100 epochs and Adamax as the optimiser with 0.001 learning rate. A detailed discussion on the experimental results related to both experiments is given in the following subsections.

### Multi-user presence

The purpose of this experiment is to determine the number of people in an indoor setting. The experiment is done in multiple phases to gradually incorporate different activities as shown in Table 4.

- In the first phase, the experiment involves only standing activity performed by a different number of subjects in parallel. The data is divided into 5 classes including the “Empty” class that represents “no-subject in the room” as shown in Table 4. For this phase of the experiment, the total number of data samples used is 557, out of which 80% (i.e. 445 samples) are randomly selected to train the model and the remaining 20% (i.e. 112 samples) are used to test the system.
- The second phase involves only the sitting activity data of all the subjects. Again, the total number of samples are 557 and the data is divided into 5 classes, 4 sitting activity classes and 1 “Empty” class.
- In the third phase, sitting and standing activity data for each number of subjects are merged to form the 4 activity classes and 1 “Empty” class. Therefore, the total number of data samples becomes 1417 out of which 80% (i.e. 1133 samples) are used to train the system while the remaining 20% (i.e. 284) samples are used for the test purpose. This data also includes the data for mixed activities like one subject sitting and one standing.
- Similarly, in the fourth phase sitting, standing, and walking data are used together to make 4 activity classes plus 1 “Empty” class. In this phase, the total number of data samples used are 1777 (1421 training samples and 356 testing samples). This data also includes mixed activity data as described previously.

**Figure 8:**
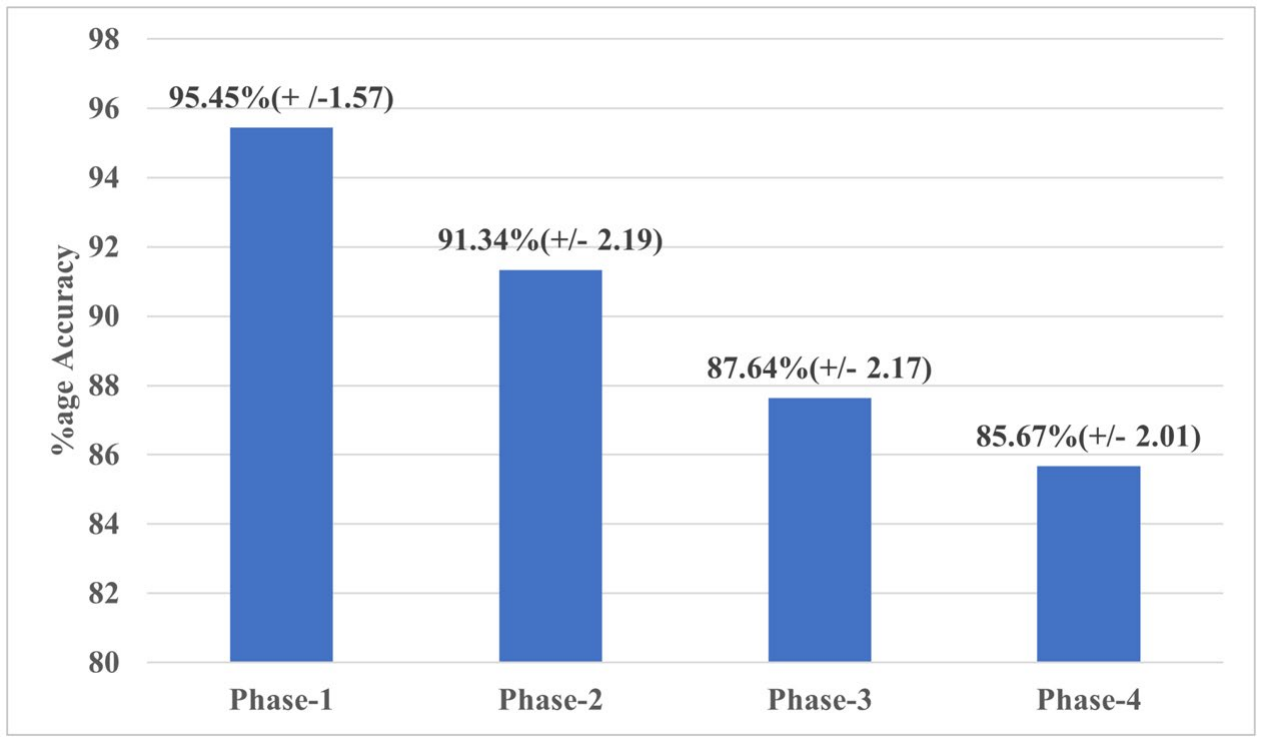
Percentage classification accuracy for all 4 phases of the subject counting experiment.

The average classification percentage accuracies for all the phases across all the 10 repetitions of each experiment are shown in the bar graph given in Fig. 8. It is observed that, in general, the proposed system works well for all the cases. However, it works better when the sitting and standing activities (Phase-1 and Phase-2) are used separately to form the activity classes. Whereas due to an increase in intra-class variation and a decrease in inter-class variation, when the sitting and standing activities are merged in Phase-3 and a new activity “walking” is introduced along with the other mixed activities in Phase-4, the overall accuracy shows a decrease.

**Figure 9:**
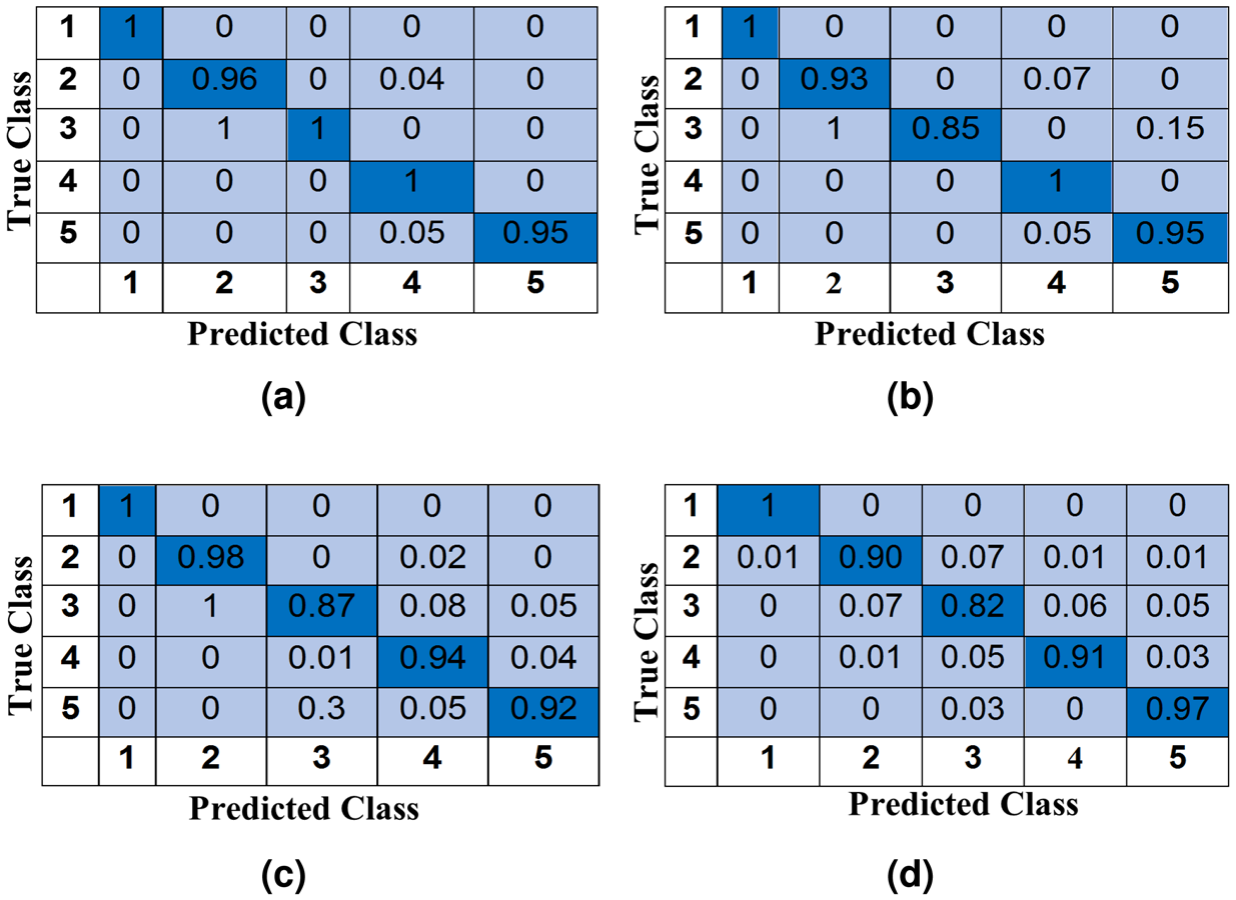
Normalised confusion matrices with maximum accuracy for all 4 phases of the experiment. The class labels for classes 1 to 5 are given in Table 4. In here (**a**) is representing Phase-1 with an accuracy of 98.22%, (**b**) is for Phase-2 with an accuracy of 94.64%, (**c**) is the Phase-3 with an accuracy of 93.31% whereas (**d**) is representing Phase-4 with an accuracy of 90.17%.

**Figure 10:**
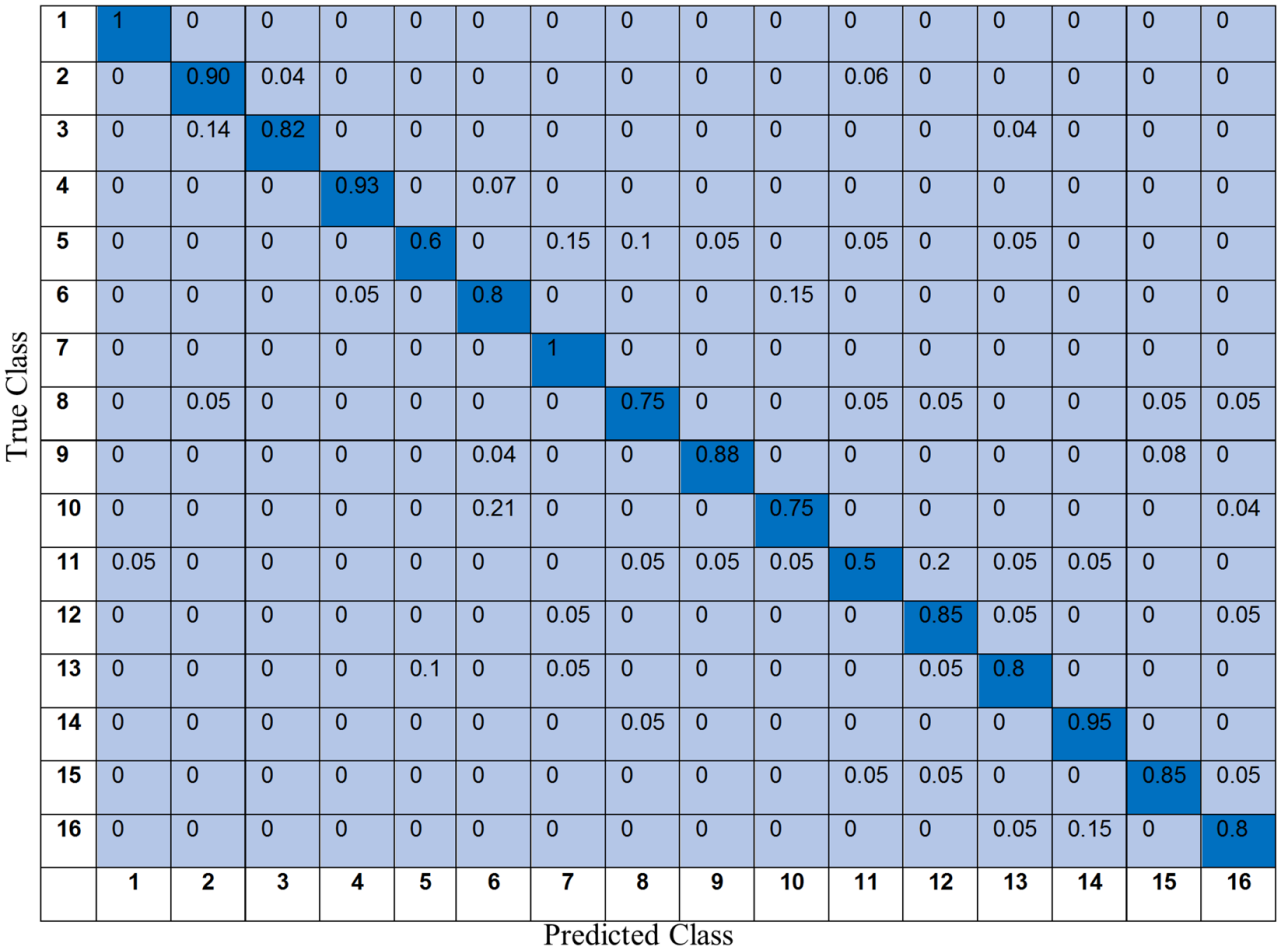
Activity monitoring: normalised confusion matrix for the fold with accuracy of 83%.

A further analysis of the confusion matrices, shown in Fig. 9, reveals that maximum misclassifications are due to 3-subject class or 4-subject class. That can be due to more subjects in a relatively smaller space that causes more noise in the USRP data. The performance can be further improved by changing the experimental environment. In this experiment, the aim is to classify human activity in one of the 16 classes given in Table 2. For this experiment, 80–20% hold-out validation is utilised and all 1777 data samples are split into training (80%) and test (20%) data and the experiment is repeated 10 times to get the average performance results. The average percentage accuracy across all 10 repetitions comes as 79.5% (± 2.6) which is very promising considering a large number of classes and lots of variation within the data. Figure 10 shows the normalised confusion matrix with the highest accuracy that is 83% for the activity recognition experiment. Here, the numbers $1,2,\ldots ,16$ are representing the 16 classes given in Table 2, respectively.

**Table 4:** Number of classes for all 4 phases for the subject count experiment.

Class number	Class labels	Phase-1	Phase-2	Phase-3	Phase-4
1	No-subject	Empty	Empty	Empty	Empty
2	1-subject	1-standing	1-sitting	1-standing/sitting	1-all activities
3	2-subject	2-standing	2-sitting	2-standing/sitting	2-all activities
4	3-subject	3-standing	3-sitting	3-standing/sitting	3-all activities
5	4-subject	4-standing	4-sitting	4-standing/sitting	4-all activities

### Activity recognition

It can be seen in the given normalised confusion matrix that in general classification performance is good for most of the activities except a few classes. The normalised confusion matrix provides the highest accuracy for empty (class 1), 1 subject sitting (class 2) and 1 subject walking (class 4) of 100%, 90% and 93%, respectively. However, there is a resemblance in CSI variations in sitting and standing activities as they are similar movements. The 1 subject standing, however, has been misclassified as standing in 14% of the samples resulting in lower accuracy of 82%. Overall, as expected the 1 subject activities provide higher accuracies due to lesser variations in CSI data as compared to multi-subject activities.

In the multi-subject case, some activities have similar patterns and are difficult to differentiate such as “1 sit + 1 stand” (class 5), “2 sitting” (class 7) and “2 standing” (class 8). Since sitting and standing movements result in quite similar motions, whether moving up or down. Also “1 sit + 1 stand” has at least 1 subject performing similar motion with “2 sitting” and “2 standing”, resulting in 15% and 10% misclassification rate of “1 sit + 1 stand” activity as “2 sitting” and “2 standing”, respectively.

Moreover, “1 walking + 1 sitting” (class 6) and “1 walking + 2 sitting” (class 10) result in a higher misclassification rate between the two activities with accuracies of 80% and 75%, respectively. As illustrated in the confusion matrix 21% of “1 walking + 2 sitting” is misclassified as “1 walking + 1 sitting” and 15% of “1 walking + 1 sitting” activity is misclassified as “1 walking + 2 sitting” activity resulting in the decrease of respective class accuracies. Similarly, “2 sit + 1 stand” (class 11) and “3 sitting” (class 12) resemble each other due to the same activities performed by at least two subjects. Furthermore, standing as mentioned before is quite similar to sitting in terms of similar motion resulting in 20% misclassification of class 11 samples as class 12. Also, similar CSI patterns exist between “4 sitting” and ”2sit + 2 stand”.

Due to the above-mentioned reasons and several classes performing sitting and standing activities with a different number of subjects, class 5 which represents two subjects (1 sitting and 1 standing) and class 11 that represents three subjects (1 sitting and 2 standing) provide the least accuracy rates of 60% and 50%, respectively in our deep learning classification model.

## Conclusions and future work

In this paper, a novel 5G-enabled contactless RF-sensing system has been presented to monitor the human presence and to detect multi-user parallel activity using CSI signals. The system’s frequency of operation was 3.75 GHz, which falls within the 3.4 to 3.8 GHz band of 5G, and to the best of the author’s knowledge, no other study has implemented 5G-sensing before. The main idea of the paper was to present a 5G-sensing based non-invasive system that is capable of detecting the presence and activities of multiple users in the same room. Furthermore, The results presented earlier in the paper have shown that by combining RF-sensing technology with standard machine learning algorithms such as CNN, it is possible to detect different human activities including counting the number of people in the room with high accuracy. The system was tested to evaluate its capability of counting as well as detect parallel activities amongst a variable number of subject, that is, between 0 and 4, as highlighted in “Multi-user presence”, respectively. The subject counting experiment reported high accuracy results between approximately 86% and 95%, with the accuracy decreasing with the increase in inter-class variations. On the other hand, the activity recognition experiment has reported approximately 80% accuracy in recognising multiple activities performed amongst all test subjects. The reported accuracy in the activity recognition experiment was greatly impacted by the intra-class variation introduced in the data. however, the variation was introduced to train the system on the maximum possible combination of input activities, to mimic a real-life scenario. The results obtained in this paper are promising and have a high potential to be improved through more data collection and the implementation of different learning algorithms. Furthermore, as the major focus of this paper is to show the significance and effectiveness of 5G-sensing in capturing variation in CSI data caused due to human activities, therefore the paper currently focuses on a small setting, such as rooms in a care home, with four persons performing three major and common activities i.e “Sitting”, “Standing” and “Walking”. In future, the aim is to scale it up to cover most of the human activity spectrum performed by a larger group in multiple rooms. Moreover, current implementation of the proposed system is based on one transmitter/receiver antenna pair and is giving better performance in comparison with most of the existing work where more than one transmitter and receiver antenna have been used for the same purpose. Moreover, and given the current system is a proof of concept with focus on showing the significance and effectiveness of 5G frequency band in capturing variation in CSI data, in future the experiments will be performed to assess the impact of number of rooms vs the number of transmitter/receiver antennas on the performance of proposed system, as well different heights and positions for the transmitter and receiver devices. Furthermore, and as mentioned earlier, the data set used to achieve the previously reported results is made publicly available, at^46^, to encourage other researchers and the wider communities to take this system a step further.

## Data Availability

The datasets generated during and/or analysed during the current study are available in the the University of Glasgow’s repository (Enlighten), and can be accessed here^46^.

## References

[1] West DM (2016). How 5g technology enables the health internet of things. Brookings Center Technol. Innov..

[2] Cook DJ, Schmitter-Edgecombe M, Dawadi P (2015). Analyzing activity behavior and movement in a naturalistic environment using smart home techniques. IEEE J. Biomed. Health Inform..

[3] Dehbandi B (2017). Using data from the microsoft kinect 2 to determine postural stability in healthy subjects: A feasibility trial. PLoS ONE.

[4] Taha A, Wu R, Emeakaroha A, Krabicka J (2018). Reduction of electricity costs in medway NHS by inducing pro-environmental behaviour using persuasive technology. Future Cities Environ..

[5] Barakat B (2021). 6G opportunities arising from internet of things use cases: A review paper. Future Internet.

[6] Taha A, Krabicka J, Wu R, Kyberd P, Adams N (2019). Design of an occupancy monitoring unit: A thermal imaging based people counting solution for socio-technical energy saving systems in hospitals. 2019 11th Computer Science and Electronic Engineering.

[7] Fabi V, Andersen RV, Corgnati SP, Olesen BW (2013). A methodology for modelling energy-related human behaviour: Application to window opening behaviour in residential buildings. Build. Simul..

[8] Calì D, Osterhage T, Streblow R, Müller D (2016). Energy performance gap in refurbished German dwellings: Lesson learned from a field test. Energy Build..

[9] Andersen RV, Toftum J, Andersen KK, Olesen BW (2009). Survey of occupant behaviour and control of indoor environment in Danish dwellings. Energy Build..

[10] Martinaitis V, Zavadskas EK, Motuziene V, Vilutiene T (2015). Importance of occupancy information when simulating energy demand of energy efficient house: A case study. Energy Build..

[11] Caicedo D, Pandharipande A, Willems FMJ (2013). Detection performance analysis of an ultrasonic presence sensor. 2013 IEEE International Conference on Acoustics, Speech and Signal Processing.

[12] Pan S (2017). Footprintid: Indoor pedestrian identification through ambient structural vibration sensing. Proc. ACM Interact. Mobile Wearable Ubiquitous Technol..

[13] Poston JD, Schloemann J, Buehrer RM, Malladi VVNS, Woolard AG, Tarazaga PA (2015). Towards indoor localization of pedestrians via smart building vibration sensing. 2015 International Conference on Localization and GNSS.

[14] Pan S, Berges M, Rodakowski J, Zhang P, Noh HY (2019). Fine-Grained recognition of activities of daily living through structural vibration and electrical sensing. Proceedings of the 6th ACM International Conference on Systems for Energy-Efficient Buildings, Cities, and Transportation.

[15] Gani MO (2019). A light weight smartphone based human activity recognition system with high accuracy. J. Netw. Comput. Appl..

[16] Taylor W (2021). Radar sensing for activity classification in elderly people exploiting micro-doppler signatures using machine learning. Sensors.

[17] Scholz M, Sigg S, Schmidkte HR, Beigl M (2011). Challenges for Device-free radio-based activity recognition. Proceedings of the 3rd Workshop on Context Systems Design Evaluation and Optimisation (CoSDEO).

[18] Woyach K, Puccinelli D, Haenggi M (2011). Sensorless sensing in wireless networks: Implementation and measurements. 2006 4th International Symposium on Modeling and Optimization in Mobile, Ad Hoc and Wireless Networks.

[19] Aggarwal JK, Ryoo MS (2011). Human activity analysis: A review. ACM Comput. Surv..

[20] Han S, Lee S (2013). A vision-based motion capture and recognition framework for behavior-based safety management. Autom. Construct..

[21] Ertin E, Stohs N, Kumar S, Raij A, Al’Absi M, Shah S, Mitra S, Kwon T, Jeong JW (2011). AutoSense: Unobtrusively wearable sensor suite for inferring the onset, causality, and consequences of stress in the field. SenSys 2011 - Proceedings of the 9th ACM Conference on Embedded Networked Sensor Systems.

[22] Yatani K, Truong KN (2011). BodyScope: A wearable acoustic sensor for activity recognition. Proceedings of the 2012 ACM Conference on Ubiquitous Computing.

[23] Xu C, Firner B, Moore RS, Zhang Y, Trappe W, Howard R, Zhang F, An N (2013). SCPL: Indoor device-free multi-subject counting and localization using radio signal strength. 2013 ACM/IEEE International Conference on Information Processing in Sensor Networks.

[24] Depatla S, Muralidharan A, Mostofi Y (2015). Occupancy estimation using only wifi power measurements. IEEE J. Select. Areas Commun..

[25] Wu FJ, Solmaz G (2016). We hear your activities through Wi-Fi signals. 2016 IEEE 3rd World Forum on Internet of Things (WF-IoT).

[26] Venkatnarayan RH, Page G, Shahzad M (2018). Multi-user gesture recognition using WiFi. Proceedings of the 16th Annual International Conference on Mobile Systems, Applications, and Services.

[27] Tan S, Zhang L, Wang Z, Yang J (2019). MultiTrack: Multi-User tracking and activity recognition using commodity WiFi. Proceedings of the 2019 CHI Conference on Human Factors in Computing Systems.

[28] Li Q (2020). Multi-user activity recognition: Challenges and opportunities. Inf. Fusion.

[29] Yang Z, Zhou Z, Liu Y (2013). From rssi to csi: Indoor localization via channel response. ACM Comput. Surv..

[30] Xi W, Zhao J, Li XY, Zhao K, Tang S, Liu X, Jiang Z (2014). Electronic frog eye: Counting crowd using WiFi. IEEE INFOCOM 2014 - IEEE Conference on Computer Communications.

[31] Feng C, Arshad S, Zhou S, Cao D, Liu Y (2019). Wi-multi: A three-phase system for multiple human activity recognition with commercial wifi devices. IEEE Internet Things J..

[32] Li J, Tu P, Wang H, Wang K, Yu L (2018). A novel device-free counting method based on channel status information. Sensors.

[33] Guo L (2019). Wiar: A public dataset for wifi-based activity recognition. IEEE Access.

[34] Yang X, Fan D, Ren A, Zhao N, Alam M (2019). 5g-based user-centric sensing at c-band. IEEE Trans. Ind. Inf..

[35] Haider D (2018). Utilizing a 5g spectrum for health care to detect the tremors and breathing activity for multiple sclerosis. Trans. Emerg. Telecommun. Technol..

[36] Tahir A (2019). Wifreeze: Multiresolution scalograms for freezing of gait detection in Parkinson’s leveraging 5g spectrum with deep learning. Electronics.

[37] Gholampooryazdi B, Sigg S (2017). Walking speed recognition from 5G Prototype System. 2017 IEEE International Conference on Pervasive Computing and Communications Workshops (PerCom Workshops).

[38] Wang Y, Liu J, Chen Y, Gruteser M, Yang J, Liu H (2014). E-Eyes: Device-Free location-oriented activity identification using fine-grained WiFi signatures. Proceedings of the 20th Annual International Conference on Mobile Computing and Networking.

[39] Wang S, Zhou G (2015). A review on radio based activity recognition. Dig. Commun. Netw..

[40] Adib F, Katabi D (2013). See through walls with wifi!. SIGCOMM Comput. Commun. Rev..

[41] Adib F, Katabi D (2013). See through Walls with WiFi!. Proceedings of the ACM SIGCOMM 2013 Conference on SIGCOMM.

[42] Pu Q, Gupta S, Gollakota S, Patel S (2013). Whole-Home gesture recognition using wireless signals. Proceedings of the 19th Annual International Conference on Mobile Computing & Networking.

[43] Taylor W (2020). An intelligent non-invasive real-time human activity recognition system for next-generation healthcare. Sensors.

[44] Ashleibta AM, Zahid A, Shah SA, Abbasi QH, Imran MA (2020). Flexible and scalable software defined radio based testbed for large scale body movement. Electronics.

[45] Abbasi QH, Abbas HT, Alomainy A, Imran MA (2021). Backscattering and RF Sensing for Future Wireless Communication.

[46] Ashleibta AMA, Taha A, Taylor W, Imran M, Abbasi Q (2021). 5g-enabled contactless multi-user presence and activity detection for independent assisted living. Res. Data.

[47] Krizhevsky A, Sutskever I, Hinton GE (2012). Imagenet classification with deep convolutional neural networks. Adv. Neural Inf. Process. Syst..

[48] Azodolmolky S (2010). Experimental demonstration of an impairment aware network planning and operation tool for transparent/translucent optical networks. J. Lightwave Technol..

[49] Kiranyaz S (2021). 1d convolutional neural networks and applications: A survey. Mech. Syst. Signal Process..

